# *Comment on* Montagna, et al. Evaluation of Legionella air contamination in healthcare facilities by different sampling methods: An Italian multicenter study. *Int. J. Environ. Res. Public Health* 2017, *14*, 670

**DOI:** 10.3390/ijerph14080876

**Published:** 2017-08-04

**Authors:** Samuel Collins, Jimmy Walker

**Affiliations:** Public Health England, Salisbury SP4 0JG, UK; jimmy.walker@phe.gov.uk

**Keywords:** *Legionella*, Legionnaires’ disease, aerosol, detection, risk assessment

## Introduction

In their recent article, Montagna et al. describe a multicenter study investigating the presence of *Legionella* in water and air samples of Italian healthcare facilities [1]. This is an interesting study that highlights some important gaps in our basic understanding of *Legionella*.

One of the limitations of the study regarded the lack of information on the tap outlets (e.g., design of tap, flow rates, temperature of the water) and the bathrooms (ambient temperature, humidity, air movements, etc.) as these factors can influence aerosols produced and may facilitate the interpretation of aerosol data. For future studies it may be advantageous to characterise the aerosols produced by these outlets, using for example an aerodynamic particle sizer (APS) [2]. Furthermore, caution should be advised as some of the conclusions from the culture data are based on the detection of just 1 colony forming unit (CFU)/m^3^ of air (or 1 CFU per hour of sampling). Comparison of this data to the number of genomic units (GU) detected in the air by Coriolis^®^ sampling is difficult, as the data is not presented as GU/m^3^. It would have been more appropriate to compare the number of GU in the water with the number of GU in the air to give a better ‘estimation’ of the emission factor. Nonetheless, the relative inability to isolate viable *Legionella* from the air is interesting and reinforces previous findings [3,4], including our own failures to culture airborne *Legionella* from both water sources [5] and compost (unpublished data). Similarly, the only successful method in these studies was liquid impingement using an all glass-cyclone sampler combined with quantitative polymerase chain reaction (qPCR).

So, why is *Legionella* difficult to culture from the air? The authors rightly state that factors such as water chemistry, the stress of aerosolisation, and the method of sampling can influence the recovery of viable *Legionella*. Furthermore, it has been shown that a high concentration of *Legionella* (>300 CFU/mL) in shower water is required to detect *Legionella* in the air [6]. This may go some way to explaining why viable airborne *Legionella* was infrequently detected at low concentrations in this study. Perhaps the low recovery of viable *Legionella* from the air is a factor in the sporadic nature of Legionnaires’ disease? However, there are also significant gaps in our understanding of *Legionella*. Quite simply, for a respiratory pathogen that is predominantly transmitted through aerosols, we have a poor understanding of *Legionella* in the aerosol state. Several key questions remain and should be research priorities in light of a globally increasing incidence of Legionnaires’ disease and an increasingly elderly and immunocompromised population:

1. What forms of *Legionella* are present in aerosols from different sources? Are they viable or infectious?
2. How is *Legionella* partitioned in the aerosol state; e.g., how much is associated with respirable particles <5 µm in size? What impact does the water system/outlet have on this?
3. What is the relationship between the amount of *Legionella* in the water and the amount of respirable *Legionella* in the air?
4. Do some *Legionella* (e.g., sequence types commonly associated with human disease) survive better in the air compared to others?
5. Do amoeba and/or amoeba cysts play a role in aerosol survival and airborne transmission of *Legionella*?
6. How far can aerosols travel in the built environment?

As the authors state, *Legionella* air sampling could inform quantitative microbial risk assessments (QMRA), improve our understanding of the risks posed by particular water systems, and inform mitigation measures. That said, there is a requirement for improved detection and characterisation of *Legionella*-containing aerosols and controlled and reproducible experiments on laboratory models and importantly, real-world systems.

Until these advances are made, it is clear that the most appropriate method for controlling the risk from *Legionella* is to control its presence in the water, eliminating the key risk factors and limiting the numbers capable of being disseminated.
